# Changing Smoking Behavior and Epigenetics: A Longitudinal Study of 4,432 Individuals From the General Population

**DOI:** 10.1016/j.chest.2022.12.036

**Published:** 2023-01-05

**Authors:** Sune Moeller Skov-Jeppesen, Camilla Jannie Kobylecki, Katja Kemp Jacobsen, Stig Egil Bojesen

**Affiliations:** a Department of Clinical Biochemistry, Herlev and Gentofte Hospital, Copenhagen University Hospital, Herlev, Denmark; b Department of Technology, Faculty of Health and Technology, University College Copenhagen, Copenhagen, Denmark; c The Copenhagen City Heart Study, Copenhagen University Hospital, Frederiksberg and Bispebjerg Hospital, Copenhagen, Denmark; d Faculty of Health and Medical Sciences, University of Copenhagen, Copenhagen, Denmark

**Keywords:** aryl hydrocarbon receptor repressor, DNA methylation, longitudinal study, lung cancer, smoking

## Abstract

**Background:**

Hypomethylation of the aryl hydrocarbon receptor repressor (*AHRR*) gene indicates long-term smoking exposure and might therefore be a monitor for smoking-induced disease risk. However, studies of individual longitudinal changes in *AHRR* methylation are sparse.

**Research Question:**

How does the recovery of *AHRR* methylation depend on change in smoking behaviors and demographic variables?

**Study Design and Methods:**

This study included 4,432 individuals from the Copenhagen City Heart Study, with baseline and follow-up blood samples and smoking information collected approximately 10 years apart. *AHRR* methylation at the cg05575921 site was measured in bisulfite-treated leukocyte DNA. Four smoking groups were defined: participants who never smoked (Never-Never), participants who formerly smoked (Former-Former), participants who quit during the study period (Current-Former), and individuals who smoked at both baseline and follow-up (Current-Current). Methylation recovery was defined as the increase in *AHRR* methylation between baseline and follow-up examination.

**Results:**

Methylation recovery was highest among participants who quit, with a median methylation recovery of 5.58% (interquartile range, 1.79; 9.15) vs 1.64% (interquartile range, –1.88; 4.96) in the Current-Current group (*P* < .0001). In individuals who quit smoking, older age was associated with lower methylation recovery (*P* < .0001). In participants who quit aged > 65 years, methylation recovery was 5.9% at 5.6 years after quitting; methylation recovery was 8.5% after 2.8 years for participants who quit aged < 55 years.

**Interpretation:**

*AHRR* methylation recovered after individuals quit smoking, and recovery was more pronounced and occurred faster in younger compared with older interim quitters.

FOR EDITORIAL COMMENT, SEE PAGE 1360

**Take-home Points**

**Study Question:** How does aryl hydrocarbon receptor repressor (*AHRR)* methylation depend on change in smoking behavior?

**Results:***AHRR* methylation is a marker of smoking behavior. It recovers years after the individual has quit smoking and is more pronounced in younger individuals.

**Interpretation:** If used as a marker of smoking-related disease risk, differential *AHRR* methylation recovery patterns should be considered.

Smoking is the primary cause of lung cancer worldwide, with 80% to 90% of all lung cancers attributable to smoking.^1^ The average life span is almost 10 years shorter in individuals who smoke compared with individuals who do not smoke, making smoking the leading cause of preventable death.^2^, ^3^, ^4^ Because objective markers for long-term smoking are lacking, quantification of tobacco consumption usually relies on self-reported smoking information; plasma cotinine and exhaled carbon monoxide reflect recent smoking behavior during the last days to weeks but cannot be used to assess smoking behavior months or years prior to examination.^5^^,^^6^ In contrast, the methylation extent of the aryl hydrocarbon receptor repressor (*AHRR*) gene has been proposed as a marker of long-term smoking.^7^^,^^8^

Smoking causes methylation changes of the *AHRR* gene, with an inverse relationship between *AHRR* methylation and smoking exposure.^9^^,^^10^ We have previously shown that *AHRR* hypomethylation correlates with current and former smoking status, cigarette consumption, cumulative smoking, smoking duration, and time since quitting smoking.^7^ *AHRR* hypomethylation has also been associated with decreased lung function and increased respiratory and cardiovascular morbidity.^11^^,^^12^ In our previous study, the risk of COPD and lung cancer was 52% and 65% higher, respectively, for every 10 percentage points lower *AHRR* methylation.^7^ Finally, lung cancer-specific mortality as well as overall mortality have been found to be elevated in individuals with low levels of *AHRR* methylation.^7^^,^^10^ However, the individual changes in methylation over time have thus far only been assessed in smaller cohorts, and if methylation status is to be implemented in clinical practice as a marker of smoking behavior and predictor of smoking-related disease risk, more information on methylation trajectories is needed. Thus, the purpose of the current study was to investigate the longitudinal change in *AHRR* methylation in a general population cohort. Specifically, we aimed to determine the change in *AHRR* methylation in blood samples taken approximately 10 years apart in four different groups characterized by their smoking history: participants who never smoked, participants who formerly smoked, participants who quit during the study period, and individuals who smoked at both baseline and follow-up. We hypothesized that the recovery of *AHRR* methylation depends on smoking behaviors and demographic variables.

## Study Design and Methods

### Study Population

We studied methylation extent of the *AHRR* gene in circulating leukocytes obtained from participants of the Copenhagen City Heart Study (CCHS). The CCHS is a Danish cohort study of the general population, initiated in the 1970s with follow-up visits every decade since. The aim of the CCHS is to describe the prevalence, incidence, and risk factors of cardiovascular and respiratory diseases. For that purpose, demographic data and questionnaires, including detailed smoking history, physical examinations, disease diagnosis, and blood samples, have been collected from every visit in the CCHS,^13^ including access to leukocyte DNA from visits in 1991 to 1994 and onward. We studied individuals' data from visits in 1991 to 1994 (ie, baseline visit) and from subsequent visits in 2001 to 2003 (ie, follow-up visit). The study was approved by Herlev and Gentofte Hospital and the Danish ethics committees (KF100.2039/91), and it was conducted according to the principles of the Declaration of Helsinki. Written informed consent was obtained from all participants.

### Methylation Measurements

The measurement technique of *AHRR* methylation is described briefly in e-Appendix 1 and was described in detail previously.^7^

### Smoking History

Data on smoking history were obtained from extensive questionnaires, completed by the participants and reviewed by the examiner at the day of attendance at baseline and at follow-up visits. Current and former smoking were defined by affirmative answers to the questions “Do you smoke?” and “If you do not smoke, have you formerly been smoking?” in the written questionnaire. Self-reported smoking of cigarettes, cheroots, cigars, and pipe tobacco were recalculated into daily grams of tobacco consumption. Cumulative smoking was calculated in pack-years for all individuals who currently or formerly smoked; a pack-year was defined as 20 cigarettes or equivalent per day smoked for 1 year. Four groups of individuals were defined based on smoking history: individuals who reported having never smoked on both visits (Never-Never), having formerly smoked on both visits (Former-Former), currently smoking at the baseline visit and having formerly smoked at the follow-up visit (Current-Former), or currently smoking at both visits (Current-Current). A subgroup of individuals (n = 146) was defined as Other because they reported other smoking trajectories (Never-Current, Former-Current, or Never-Former).

### Covariates

Covariates were considered a priori to reflect general lifestyle and health of the individuals (alcohol intake, education, including BMI, systolic BP, lung function, plasma C-reactive protein, COPD, and diabetes mellitus). These are described in detail in e-Appendix 1.

### Statistical Analysis

Statistical analysis was performed by using Stata version 15.1 for Microsoft Windows (StataCorp). Methylation extent was compared between baseline and follow-up by using paired *t* tests. Between-group differences were analyzed by using the χ^2^ test or one-way analysis of variance with post hoc Bonferroni corrections. Linear regression models were used to predict the absolute methylation recovery in the Current-Former group. The assumption of linearity was assessed visually by using a plot of observed vs predicted values. Homoscedasticity was tested for visually by using a plot of residuals vs predicted values, and for normality of residuals plotting quintiles of the variable against quintiles of the normal distribution. No major violations of linearity, homoscedasticity, or normality were observed. Factors included in the linear regression model were selected based on a model reduction approach with backward elimination of nonsignificant covariables. The first, nonreduced model included the variables age, BMI, alcohol consumption, educational level, C-reactive protein, FEV to FEV_1_ ratio, systolic BP, baseline *AHRR* methylation, smoking intensity, and years since quitting smoking (all on a continuous scale), as well as sex, exposure to dust and secondhand smoke, diagnosis of COPD, and diagnosis of diabetes (all on a dichotomized scale). The final, reduced model included the variables sex, age, smoking intensity (cigarettes per day), years since quitting, educational level, and baseline *AHRR* methylation as determinants for the *AHRR* methylation recovery. Because of the nonlinear relationship between methylation recovery and years since quitting, a restricted cubic spline was used to optimize the fit of the regression.^14^ A linear regression model and restricted cubic spline, as described earlier, were used to assess and depict the association between smoking intensity and methylation at baseline and follow-up.

## Results

In the current study, 16,560 individuals were invited for the baseline examination and 10,135 individuals attended (57% response rate). At baseline, 9,432 individuals had blood drawn, and 9,234 had methylation measured successfully. At the follow-up visit, 4,766 individuals attended (52% response rate) and 4,432 had methylation measurement repeated. Of these, 1,313 individuals (29%) reported to never smoke at both examinations (Never-Never group); 1,144 individuals (25%) reported to having formerly smoked at both examinations (Former-Former group); 575 individuals (13%) reported currently smoking but having quit smoking between examinations (Current-Former group); and 1,400 individuals (31%) reported currently smoking at both examinations (Current-Current group) (Table 1). Individuals with other smoking trajectories (n = 146) were excluded from the study. The smoking trajectories for individuals with failed methylation measurement were comparable to the smoking trajectories for the individuals with repeated measurements. There were 4,468 individuals who did not participate in the follow-up examination. These individuals were generally characterized by a less favorable profile such as older age, higher cumulative smoking, more comorbidities, and higher C-reactive protein levels compared with individuals who did participate in the follow-up examination.

**Table 1:** Participant Characteristics at Baseline

Characteristic	Included in Study				Excluded From Study		
	Never-Never	Former-Former	Current-Former	Current-Current	Other^a^	Failed Methylation Measurement^b^	Not Participating in Follow-up
Individuals, No.	1,313	1,144	575	1,400	146	386	4,468
Methylation, %	64.2 (60.4; 68.0)	60.4 (55.6; 65.0)	51.2 (48.1; 56.3)	50.0 (47.1; 53.3)	59.4 (53.6; 64.1)	...	54.2 (49.1; 61.3)
Male	449 (34.2)	548 (47.9)	268 (46.6)	607 (43.4)	70 (48.0)	139 (36.0)	2,105 (47.1)
Age, y	53.2 (39.5; 64.0)	58.7 (47.2; 67.0)	54.5 (42.5; 63.4)	53.9 (44.0; 61.4)	50.6 (37.8; 61.0)	56.2 (43.3; 68.3)	66.7 (54.8; 74.0)
Cumulative smoking, pack-y^c^	0.0 (0.0; 0.0)	10.5 (3.8; 24.0)	18.6 (9.0; 31.2)	25.7 (15.0; 37.5)	3.0 (0.0; 17.0)	8.0 (0.0; 26.3)	20.0 (1.5; 38.4)
Smoking intensity, cigarettes per d	0 (0; 0)	0 (0; 0)	13 (7; 20)	15 (10; 20)	8 (0; 16)	10 (0; 20)	12 (2; 20)
BMI, kg/m^2^	24.8 (22.4; 27.8)	25.4 (23.1; 27.9)	24.4 (22.2; 26.8)	24.0 (22.1; 26.8)	25.1 (22.4; 27.9)	24.9 (22.1; 28.5)	25.2 (22.6; 28.4)
Alcohol, units/wk^d^	4 (1; 9)	6 (2; 12)	7 (2; 14)	7 (2; 16)	7 (3; 15)	5 (0; 11)	5 (0; 13)
Education, y	10 (8; 12)	10 (7; 12)	9 (7; 12)	9 (7; 11)	10 (8; 12)	9 (7; 12)	8 (7; 10)
Systolic BP, mm Hg	130 (118; 145)	135 (121; 151)	130 (120; 143)	130 (119; 142)	132 (120; 145)	135 (120; 152)	140 (126; 158)
COPD	2 (0.2)	9 (0.8)	3 (0.5)	4 (0.3)	0 (0.0)	3 (0.8)	131 (2,9)
Diabetes	31 (2.4)	31 (2.7)	11 (1.9)	25 (1.8)	1 (0.7)	14 (3.6)	298 (6.7)
CRP, mg/L	1.45 (1.14; 2.14)	1.52 (1.18; 2.37)	1.58 (1.21; 2.51)	1.65 (1.25; 2.79)	1.47 (1.15; 2.22)	1.65 (1.17; 2.74)	1.99 (1.35; 3.64)
Lung function, FEV_1_/FVC ratio	0.82 (0.78; 0.86)	0.80 (0.75; 0.84)	0.79 (0.73; 0.84)	0.78 (0.73; 0.83)	0.80 (0.76; 0.84)	0.80 (0.75; 0.84)	0.77 (0.70; 0.82)

Data are presented as median (25 percentile; 75 percentile) for continuous variables or as No. (%) for categorical variables. CRP = C-reactive protein.

a Other: Never-Current, Former-Current, Never-Former.

b Individuals with failed methylation measurement at baseline (n = 198) or follow-up (n = 188).

c One pack year = 20 cigarettes per day over 1 year.

d 1 unit = 12 g alcohol.

### Methylation Changes From Baseline to Follow-up

**Figure 1:**
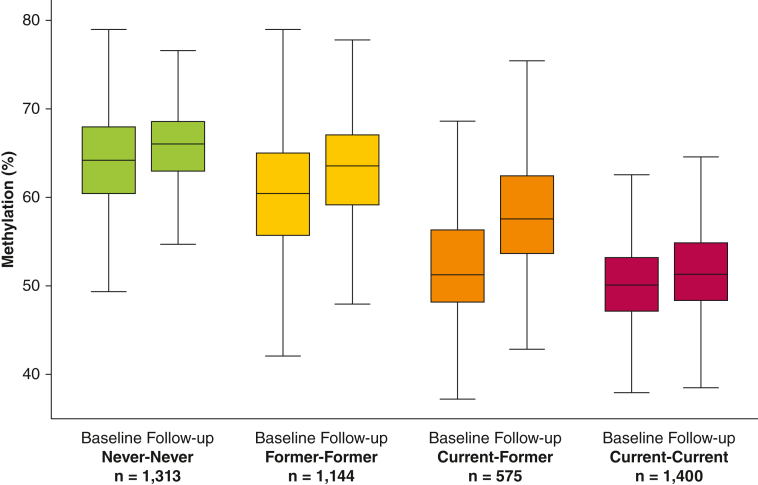
Methylation extent at baseline and follow-up stratified according to smoking history. Boxplots showing the absolute methylation extent at baseline and follow-up examinations in four smoking history groups. Outliers are not shown. Never: individuals who never smoked at the time of the baseline/follow-up examination. Former: individuals who formerly smoked at the time of the baseline/follow-up examination. Current: individuals who smoked at the time of the baseline/follow-up examination.

**Figure 2:**
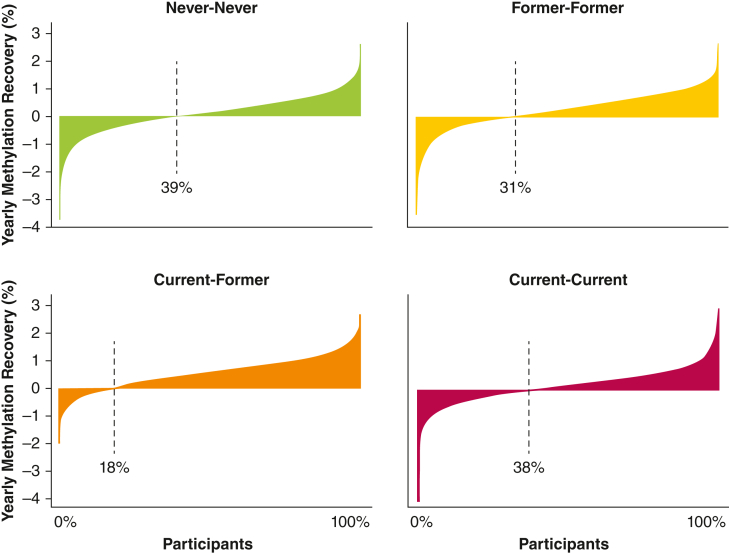
Smoking history and yearly methylation recovery. Waterfall plots showing the yearly methylation recovery in each individual, stratified according to smoking behavior groups (Never-Never = individuals who never smoked; Former-Former = individuals who formerly smoked; Current-Former = individuals who quit during the study period; and Current-Current = individuals who smoked at both baseline and follow-up). Dashed line: percentage of individuals with negative methylation recovery at follow-up. Absolute methylation recovery is shown in percentage points per year on the y-axis.

At baseline, the median methylation extent was 64.2% (interquartile range [IQR], 60.4; 68.0) in the Never-Never group compared with 60.4% (IQR, 55.6; 65.0) in the Former-Former group, 51.2% (IQR, 48.1; 56.3) in the Current-Former group, and 50.0% (IQR, 47.1; 53.3) in the Current-Current group (overall, *P* < .001; post hoc between-group comparisons, *P* < .001, for all combinations). As expected, these declining median methylation extents were paralleled by higher median cumulative smoking (0, 10.5, 18.6, and 25.7 pack-years, respectively) (Table 1). From baseline to follow-up, the median methylation extent increased by 1.34% (IQR, –2.42; 5.25) in the Never-Never group, 2.84% (IQR, –1.02; 6.56) in the Former-Former group, 5.58% (IQR, 1.79; 9.15) in the Current-Former group, and 1.64% (IQR, –1.88; 4.96) in the Current-Current group (overall, *P* < .001; post hoc between-group comparison Never-Never vs Current-Current, *P* = .18; Former-Former vs Current-Current, *P* = .004; all other combinations, *P* < .001) (Fig 1). At follow-up, the methylation extent was still significantly different between the groups (overall, *P* < .001; post hoc between-group comparison, *P <* .001 for all combinations). In the Current-Former group, only 18% of the individuals had lower methylation at follow-up than at baseline, whereas the corresponding percentages were 39% in the Never-Never group, 31% in the Former-Former group, and 38% in the Current-Current group (*P* < .001) (Fig 2).

### Linear Regression Analysis of the Association Between Methylation and Smoking Intensity

**e-Figure 1:**
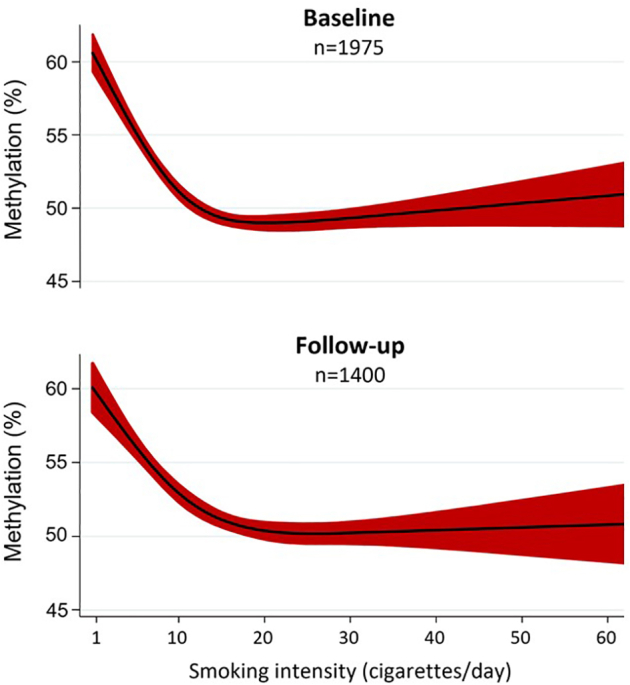


In individuals who smoked at baseline or follow-up, greater smoking intensity was associated with lower methylation level in a nonlinear model (e-Fig 1). The mean methylation level decreased from approximately 60% in individuals smoking 1 cigarette per day to approximately 50% in individuals smoking 20 cigarettes per day. In individuals smoking > 20 cigarettes per day, the methylation level seemed to remain constant at approximately 50% independent of additional smoking intensity.

### Linear Regression Analysis of the Association Between Methylation Recovery and Age

**Figure 3:**
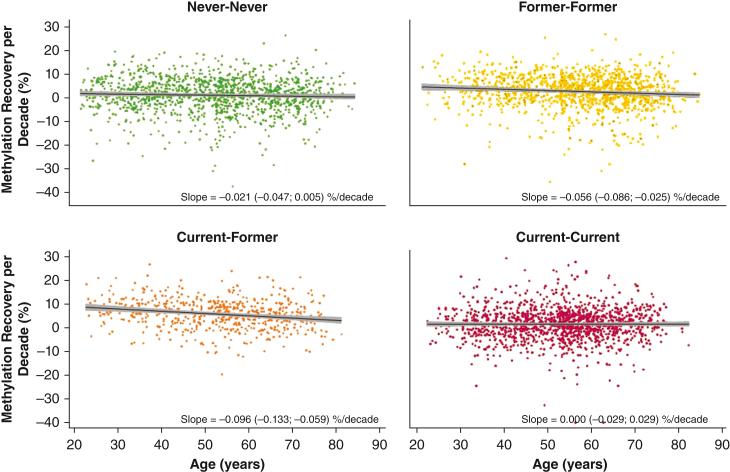
Methylation recovery as a function of age. Scatter plots showing the methylation recovery per decade in four smoking history groups (Never-Never = individuals who never smoked; Former-Former = individuals who formerly smoked; Current-Former = individuals who quit during the study period; and Current-Current = individuals who smoked at both baseline and follow-up). Fitted lines with 95% CIs were based on unadjusted linear regression, with age at baseline in years as an independent variable and methylation recovery per decade as a dependent variable. Absolute methylation recovery per decade is shown on the y-axis in percentage points.

In the Former-Former group and the Current-Former group, older vs younger age at baseline was associated with lesser methylation recovery during follow-up in unadjusted linear regression models (Fig 3). In the Former-Former group, methylation recovery per decade was 0.056% (95% CI, 0.086-0.025; *P <* .001) lower per 1-year increase in age at baseline. Likewise, in the Current-Former group, methylation recovery per decade was 0.096% (95% CI, 0.133-0.059; *P* < .001) lower per 1-year increase in age at baseline. Thus, an interim quitter aged 30 years would have 4.8% additional methylation recovery over a 10-year period compared with an interim quitter aged 80 years. In the Never-Never group and the Current-Current group, age at baseline was not associated with methylation recovery.

### *AHRR* Methylation Recovery in Individuals Who Quit During Follow-up

**Figure 4:**
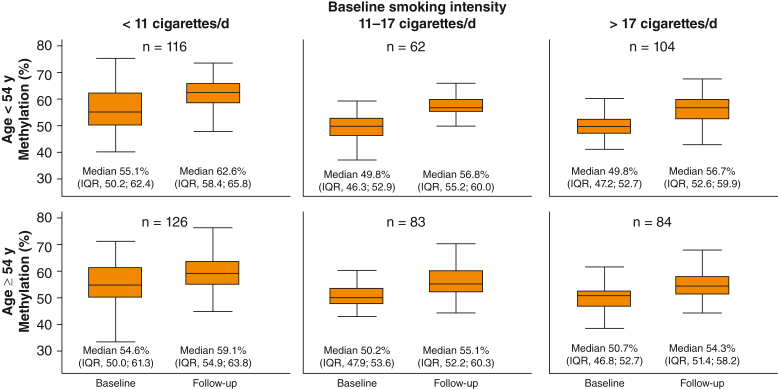
Methylation development after quitting. Boxplots showing the methylation extent at baseline and follow-up for individuals in the Current-Former group (interim quitters). Individuals were stratified in six groups based on median age at baseline (54 y) and smoking intensity tertiles at baseline (< 11 cigarettes per day, 11-17 cigarettes per day, and > 17 cigarettes per day). Extent of methylation is shown on the y-axis in percentage points.

Median and IQR values in the Current-Former group were stratified on median age and tertiles of smoking intensity at baseline (Fig 4). In individuals aged < 54 years who smoked < 11 cigarettes per day, the median methylation was 55.1% (IQR, 50.2; 62.4) at baseline and 62.6% (IQR; 58.4; 65.8) at follow-up; the corresponding medians for individuals who smoked > 17 cigarettes per day were 49.8% (IQR, 47.2; 52.7) and 56.7% (IQR, 52.6; 59.9). In individuals aged ≥ 54 years, recovery was less, in general, than in the younger age groups. However, in individuals aged ≥ 54 years, methylation was still statistically significantly higher at follow-up compared to baseline.

**Figure 5:**
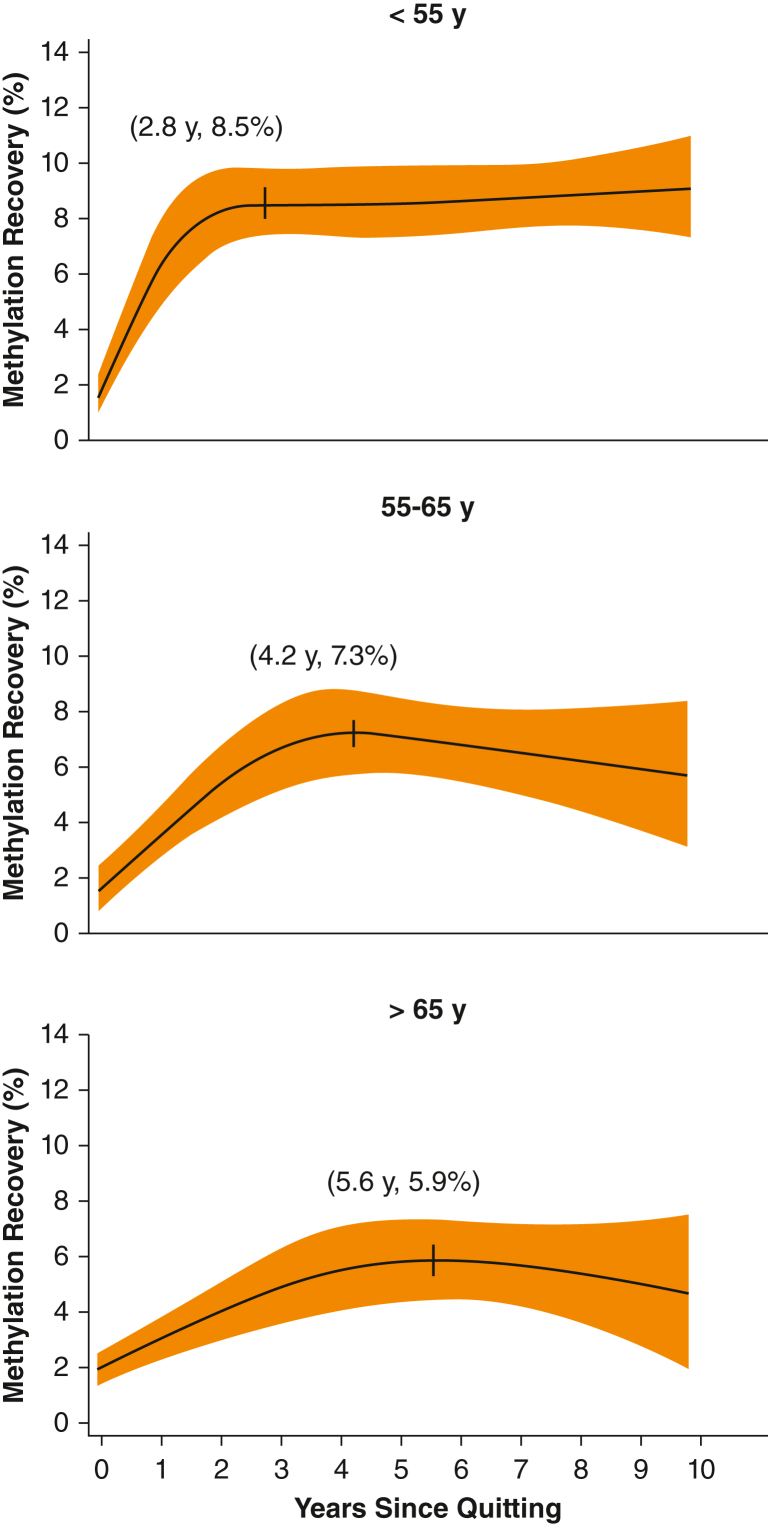
Modeled methylation recovery after quitting. Methylation recovery as a function of years since self-reported smoking quitting in the Current-Former group (interim quitters). The model was adjusted for sex, smoking intensity (cigarettes per day), years since quitting, educational level, and baseline aryl hydrocarbon receptor repressor methylation. Individuals were stratified into three age groups based on age at quitting. Methylation recovery in shown on the y-axis in percentage points. Marginal effects function was used to determine the top coordinates on the curves.

We next modeled *AHRR* methylation recovery depending on the time since quitting. Among the 575 Current-Former individuals, male sex, older age, low educational level, and greater smoking intensity were associated with lower methylation recovery after quitting (*R*^2^ = 0.37; *P* < .0001). A nonlinear relationship was found between methylation recovery and years since quitting. Depending on age at quitting, the methylation recovery seemed to level out 3 to 6 years after quitting (Fig 5). In individuals aged < 55 years, we found an absolute increase of 8.5% methylation within 2.8 years after quitting, after which the recovery leveled out. Similar estimates in individuals aged 55 to 65 years and > 65 years were 7.3% after 4.2 years and 5.9% after 5.6 years.

## Discussion

In this study of 4,432 individuals with repeated methylation measurements, methylation at the cg05575921 site in the *AHRR* gene recovered to a greater degree in individuals who quit compared with individuals who continued smoking or individuals who never smoked. Furthermore, we found that male sex, older age, low educational level, and greater smoking intensity were associated with lower methylation recovery after quitting. Finally, methylation recovery occurred faster and to a higher degree following quitting in younger individuals than in older individuals. These are novel findings.

Previous studies of methylation changes over time have been equivocal. One study found that the recovery of DNA methylation in participants who formerly smoked stabilized 5 years after smoking cessation independent of previous smoking intensities.^15^ Other studies found that DNA methylation recovery continued for 2 to 4 decades following smoking cessation.^16^, ^17^, ^18^ In contrast, we found that *AHRR* methylation recovery stabilized 3 to 6 years after smoking cessation depending on age. As found in the current study, another recent study found recovery of *AHRR* methylation in interim quitters^19^; however, in contrast to the current study, time since quitting was not evaluated. More short-term studies have found rapid *AHRR* methylation recovery within months of smoking cessation^20^^,^^21^; in comparison, the current study showed lesser *AHRR* methylation recovery within the first year after smoking cessation and that the rate of *AHRR* recovery after smoking cessation depended on age. Notably, 18% of individuals who quit smoking during follow-up in the current study had lower *AHRR* methylation at follow-up than at baseline despite smoking cessation, thus showing the influence of known and unknown confounders on the association between methylation and smoking behaviors.

The mechanisms linking hypomethylation, smoking, and lung disease are not well described, but inflammatory processes may be implicated as *AHRR* hypomethylation is associated with the activation of natural killer cells.^22^ Furthermore, macrophages obtained by BAL in individuals who smoke exhibit lower *AHRR* methylation along with altered methylation of genes involved in inflammatory signaling pathways.^23^ Increased expression of *AHRR* leads to lower activity of the aryl hydrocarbon receptor, which regulates xenobiotic metabolism through cytochrome P450.^24^ It has been reported that as much as 32% of the smoking-induced risk for lung cancer may be mediated through *AHRR* hypomethylation, and *AHRR* is downregulated in several types of malignancies, including lung cancer.^25^^,^^26^ However, an epidemiologic study using Mendelian randomization found no evidence of a causal relationship between *AHRR* hypomethylation and lung cancer.^27^ Despite inconsistent evidence regarding biological pathways and causality, the predictive value of *AHRR* methylation in relation to lung cancer has been shown in several cohorts.^7^^,^^9^^,^^25^^,^^27^, ^28^, ^29^

In terms of the observed difference in methylation recovery in younger vs older subjects, this could be explained by an altered function of DNA methyltransferases (DNMTs) in older individuals. Different DNMTs are known to regulate methylation; DNMT1 copies DNA methylation patterns during cell replication, and DNMT3a and DNMT3b introduce de novo methylation of CpG sites.^30^^,^^31^ Aging is associated with decreased activity of DNMTs and overall hypomethylation of the genome.^32^^,^^33^ Although the precise mechanisms underlying these age-related changes are unknown, previous studies show that oxidative stress and inflammation affect the localization of DNMTs and their binding to DNA.^34^^,^^35^ Age-related low-grade systemic inflammation and oxidative stress may influence methylation recovery,^36^, ^37^, ^38^ as observed in the current study.

Several strengths and limitations of the current study should be addressed. Strengths of this study include the large study size, which, to the best of our knowledge, represents the largest cohort with repeated measurements of *AHRR* methylation; the detailed smoking history; and the large number of included confounders. However, the following limitations should be considered.

Generalizability: Our study was performed in a Danish population, which might limit the generalizability of the results in respect to other ethnic populations. However, we are not aware of data suggesting that results would have differed in other ethnicities.

Nonattendance to follow-up: Only 52% of individuals who participated in the baseline visit returned to the follow-up visit approximately 10 years later. In general, individuals who did not attend follow-up were older, had more comorbidities, and had a lower educational level compared with individuals who did attend follow-up. Because these covariates were associated with lower *AHRR* methylation recovery, this nonparticipation may have biased our results.

Reporting of smoking status: Because smoking information was obtained from questionnaires, and plasma cotinine levels were not available to validate this information, it is possible that underreporting of tobacco use, as is usually suspected in epidemiologic studies,^39^ may have biased our results toward the null hypothesis. Thus, the association between smoking intensity, time since quitting, and *AHRR* methylation may be even more pronounced than captured in the current study. However, it should also be mentioned that there were no incentives, other than purely scientific, for individuals to participate in this study, and no health insurance or commercial benefits or drawbacks associated with reporting of smoking status.

Methodologic aspects: A number of different methods have been used in previous studies to measure *AHRR* methylation: methylation arrays,^9^^,^^10^ digital polymerase chain reaction,^40^^,^^41^ pyrosequencing,^42^ mass array,^43^ and quantitative polymerase chain reaction.^20^ We analyzed *AHRR* methylation by using a TaqMan-based assay, which is relatively inexpensive and convenient for large sample sizes. We previously showed that the measurements from the TaqMan-based assay were highly correlated with measurements obtained from pyrosequencing (*R*^2^ = 0.70; F = 381; *P* < .0001).^7^ The mean *AHRR* methylation level in individuals who do not smoke has previously been reported to be > 80%,^8^^,^^44^ which is significantly higher than the methylation level in participants who never smoked in the current study. Because the numerical values of *AHRR* methylation and methylation recovery may differ depending on which assay is used, results from different assays may not be directly comparable across studies, although still valid within each study. Evidently, standardization of methylation measurement methods is needed prior to application of *AHRR* methylation for clinical purposes.

*AHRR* methylation recovery in participants who never smoked: The current study found a small, but unexpected, recovery of *AHRR* methylation in participants who never smoked, which differs from previous findings of near constant methylation levels in individuals who never smoked.^16^ Several possible explanations to these findings exist. First, because baseline samples and follow-up samples were not analyzed in random order at the same time point, the observed shift in methylation among participants who never smoked could be due to slight batch variation in the assay used to measure *AHRR* methylation. However, this bias would be independent of smoking categories and thus does not invalidate the observed differences in methylation changes between smoking categories. Second, it is possible that participants who never smoked were exposed to less secondhand smoke and air pollution during follow-up due to general trends in the Danish society during the 1990s, and that our measurements reflect a true recovery of *AHRR* methylation in individuals who never smoked. Finally, we cannot exclude the possibility that the Never-Never group included some individuals who smoked at baseline and quit smoking during follow-up; however, this misclassification is unlikely to explain all the observed recovery.

## Interpretation

Future studies may address the additional value of *AHRR* methylation recovery in predicting risks of pulmonary diseases. A single measurement of *AHRR* methylation correlates with the risks of COPD, lung cancer, ischemic heart disease, and death.^7^^,^^10^, ^11^, ^12^ Consequently, risk assessment based on measured methylation extent may underestimate smoking burden if measured years after quitting, especially in younger individuals. Conversely, if DNA methylation is on the causal pathway between smoking and disease, the observed methylation recovery may suggest a mechanism by which smoking damage can be offset following smoking cessation. These dynamics may need to be captured in future studies of *AHRR* methylation.

We found that methylation at the cg05575921 site in the *AHRR* gene recovered to a greater degree in individuals who quit smoking compared with individuals who continued smoking or who never smoked. Also, methylation recovery occurred faster and to a greater degree in quitters of younger than older age. Future studies may assess the value of longitudinal changes in *AHRR* methylation in relation to clinical outcomes.

## Funding/Support

The study was funded by the 10.13039/501100002918Department of Clinical Biochemistry, Herlev and Gentofte Hospital, Copenhagen University Hospital, and the Independent Research Fund Denmark.

## Financial/Nonfinancial Disclosures

None declared.

## Supplementary Data

e-Online Data
