# Supplementary material for: Changing Smoking Behavior and Epigenetics: A Longitudinal Study of 4,432 Individuals From the General Population
Source: Chest. 2023 Jan 5;163(6):1565–75. doi: 10.1016/j.chest.2022.12.036 (PMC10258440; doi:10.1016/j.chest.2022.12.036)
Supplement: e-Online Data [file mmc1.docx]

e-Appendix 1

**Changing smoking behavior and epigenetics: a longitudinal study of 4,432 individuals from the general population**

Sune Moeller Skov-Jeppesen^1^, Camilla Jannie Kobylecki^1^, Katja Kemp Jacobsen^2^, Stig Egil Bojesen^1,3,4,^*

^1^Department of Clinical Biochemistry, Herlev and Gentofte Hospital, Copenhagen University Hospital, Herlev, Denmark.

^2^Department of Technology, Faculty of Health and Technology, University College Copenhagen, Copenhagen, Denmark.

^3^The Copenhagen City Heart Study, Copenhagen University Hospital, Frederiksberg and Bispebjerg Hospital, Denmark.

^4^Faculty of Health and Medical Sciences, University of Copenhagen, Copenhagen, Denmark.

*Corresponding author: Stig Egil Bojesen, Department of Clinical Biochemistry, Herlev and Gentofte Hospital, Copenhagen University Hospital, Borgmester Ib Juuls Vej 1, 2730 Herlev, Denmark. E-mail: stig.egil.bojesen@regionh.dk

Table of contents Page No.

Methylation measurement 2

Covariates 2

***Methylation measurements***

In short, blood samples were drawn at baseline and follow-up examination. Isolated leukocyte DNA was treated with bisulphite to convert unmethylated cytosine to uracil (thymine after PCR amplification). The methylation extent was measured at a single CpG site, cg05575921, located in the intron 3 region of the *AHRR* gene on chromosome 5. Amounts of methylated and unmethylated cytosine at cg05575921 were determined using a TaqMan assay with two different probes binding to either the non-converted cytosine residue or the converted thymine residue and methylation extent in percentage was calculated. Duplicate measurements were performed for each sample. Analyses were carried out on plates containing duplicates of 11 standard samples of DNA with methylation extent split by 10% intervals and ranging from 0-100%. Coefficients of variation at 71% methylation extent varied from 5.0 to 6.7% for different lots of the internal control measured in all plates. Measurements were adjusted for batches of standard samples and validated using pyrosequencing of 170 samples. *AHRR* methylation extent is reported as percentage ranging from 0 to 100 with *AHRR* methylation recovery reported as absolute changes in percentage points (percentage at follow-up minus percentage at baseline).

***Covariates***

Information on alcohol intake and education was self-reported. Body mass index was calculated as measured weight in kilograms divided by measured height in meters squared. Systolic blood pressure was measured at time of examination. Forced expiratory volume in 1 second (FEV1) and forced vital capacity (FVC) were determined using a dry wedge spirometer (Vitalograph; Maids Moreton, Buckinghamshire) and spirometry was performed in triplicate and the average of the two best performances reported. C-reactive protein (CRP) was measured with a high-sensitivity standard hospital assay. Diagnoses of chronic obstructive pulmonary disease (COPD) were drawn from the Danish National Patient Register (DNPR). Diabetes mellitus (DM) was any type of DM reported in questionnaire or registered in DNPR, use of antidiabetic treatment, or plasma glucose >11 mmol/L at day if attendance.
